# The impact of prior online gaming experience on the migration of offline gamblers to online gambling platforms

**DOI:** 10.1371/journal.pone.0331451

**Published:** 2025-09-05

**Authors:** Young-Hee Ko, Hohyun Kim

**Affiliations:** 1 Department of Business Administration, Seoul Business School, aSSIST University, Seoul, Republic of Korea; 2 Department of Advanced Industry Fusion, Konkuk University, Seoul, Republic of Korea; Yale University, UNITED STATES OF AMERICA

## Abstract

The proliferation of online gambling platforms has heightened concerns over their potential to intensify problematic gambling behaviors. While previous research has examined various risk factors, the influence of prior online gaming experience on gambling transitions remains underexplored. This study investigates whether and how engagement with online gaming facilitates the migration from offline to online gambling. Using survey data from 742 adults in South Korea, the analysis demonstrates that individuals with prior online gaming experience are significantly more likely to engage in online gambling, place bets more frequently, and spend larger amounts on real-money online betting. Among this group, higher expenditures on in-game purchases are positively associated with increased gambling frequency and spending, indicating a behavioral link between financial investment in online gaming and subsequent online gambling behavior. The study further identifies two prominent features of online gambling—platform anonymity and the perceived advantage of data-driven decision-making—as especially appealing to experienced online gamers. These findings reveal key psychological and behavioral mechanisms that connect online gaming to online gambling engagement. They also underscore the need for regulatory frameworks that address the evolving risks posed by digitally mediated gambling environments.

## Introduction

The expansion of online gambling has become a significant topic of discussion within the gambling industry, particularly in the wake of the COVID-19 pandemic. As brick-and-mortar casinos and betting venues experienced steep revenue declines due to lockdowns and social distancing measures, many regulated gambling operators sought to mitigate financial losses by advocating for the legalization and rapid development of online betting services [1]. This shift has been accompanied by major policy reforms in several jurisdictions. For example, mobile sports betting was legalized in Arizona and New York in 2021, and over 30 U.S. states allow online sports betting in 2025. Ontario launched a regulated online casino market in 2022, and South Korea approved online horseracing betting in June 2024. These developments signal a global trend toward the digitalization of gambling markets, with market forecasts predicting continued growth in the years ahead.

While the expansion of online gambling brings potential economic benefits and fosters innovation and accessibility, it also presents pressing public health concerns. Research consistently links online gambling with elevated risks of problem gambling, particularly due to its structural features—such as unrestricted access, anonymity, and the use of persuasive digital interfaces—that distinguish it from land-based gambling [2,3]. Unlike traditional gambling venues that require physical presence and are subject to operating hours and regulatory controls, online platforms offer continuous availability, enabling users to place bets at any time and from virtually any location. This convenience, combined with the perceived privacy of online environments, may contribute to increased gambling frequency, greater financial expenditure, and a heightened risk of gambling-related harm [4–8]. Previous literature suggests that young adults, particularly males, are disproportionately engaged in online gambling and are more susceptible to its negative consequences, including problem gambling and financial harm [9–11]. Moreover, individuals experiencing psychological distress, loneliness, or lower socioeconomic status have been found to be at heightened risk for problematic online gambling behaviors [12,13]. Building on these findings, this study attempts to identify a behavioral, individual-level factor that may influence susceptibility to online gambling engagement.

A growing but still underexplored factor in this domain is prior experience with online video gaming (hereafter, online gaming). As digital entertainment continues to evolve, many online games now incorporate monetization mechanisms that bear striking structural and psychological similarities to gambling. These include microtransactions, loot boxes, randomized rewards, and in-game betting systems, which collectively blur the boundaries between gaming and gambling [14–17]. Exposure to these features may foster behavioral conditioning toward financial risk-taking and reinforce reward-driven engagement, potentially priming individuals for gambling behaviors. Empirical studies have found that gaming mechanics can shape gambling-related attitudes and behaviors, increasing susceptibility to gambling initiation and persistence [18–20].

Despite these insights, few studies have directly examined the behavioral transition from online gaming to online gambling, particularly among individuals who previously participated in offline gambling. Individuals with extensive gaming experience may face fewer psychological and technological barriers when engaging with online gambling platforms [21]. Both environments share core features—such as anonymity, interactivity, and real-time decision-making—that may facilitate behavioral continuity. Moreover, familiarity with data-driven gaming strategies, such as analyzing probabilities and optimizing in-game purchases, may transfer to online gambling contexts, where similar skills are perceived to improve betting outcomes. Notably, contemporary video games often incorporate gambling-like mechanics that can lower the barrier to actual gambling [22,23]. Understanding the potential influence of online gaming on online gambling migration is therefore essential for identifying emerging risk factors and informing evidence-based policy interventions aimed at reducing problematic gambling behaviors and associated harms.

### Online gambling and online gaming environments in South Korea

In South Korea, the gambling industry has historically operated under stringent regulatory constraints, with most forms of online gambling remaining illegal [24,25]. While online sports betting through government-authorized channels has been permitted since 2001, other gambling activities such as online casino gaming and cow fighting continue to be banned. However, the COVID-19 pandemic marked a turning point in national gambling policy, as traditional gambling venues suffered substantial revenue losses due to mandatory lockdowns and distancing regulations [26]. In an effort to curb the rise of illegal offshore gambling and recapture domestic revenue, South Korea began reconsidering its stance on online gambling platforms. This led to the legalization of online betting for boat and bicycle racing in 2021 [27], followed by the approval of online horse race betting in 2024. These policy shifts represent a significant departure from previous restrictions and indicate a broader willingness to regulate, rather than prohibit, online gambling activity.

Despite these developments, the expansion of online gambling in South Korea remains a contentious issue. Advocates of legalization argue that a regulated online market can enhance transparency, support responsible gambling initiatives, and generate substantial tax revenue [28]. Proponents further emphasize that legal platforms provide an opportunity to implement safeguards that are absent in unregulated markets, including player verification systems, deposit limits, and early detection tools for problematic gambling behavior [29–32]. In contrast, critics caution that the digital nature of online gambling—particularly its anonymity, constant accessibility, and immersive interface—can exacerbate gambling-related harm, especially among younger and vulnerable populations [33]. These divergent perspectives underscore the importance of understanding the behavioral drivers behind online gambling participation. In particular, identifying how individuals transition from offline to online gambling is critical to designing evidence-based policies and harm reduction strategies tailored to the evolving gambling environment.

Furthermore, South Korea offers a particularly relevant context for investigating whether and how online gaming experience influences the migration of offline gamblers to online gambling platforms. According to 2023 estimates by the International Telecommunication Union, 97% of Korea’s population uses the Internet—placing the country among the top 12 globally—and it also has a deeply entrenched online gaming culture [34]. The country has also witnessed growing public debate and regulatory scrutiny over monetization strategies in online games—particularly loot boxes—due to concerns that such mechanics may normalize high-risk financial behavior among young users [35]. These characteristics make South Korea a valuable empirical setting for investigating the behavioral pathways that connect online gaming and gambling.

### Aim of study

This study aims to examine whether and how prior online gaming experience influences the transition of offline gamblers to online gambling platforms. By identifying this behavioral linkage, we attempt to contribute to the growing literature on the digitalization of gambling and provide insights for designing targeted harm-reduction policies. Findings may also offer practical implications for regulators and policymakers navigating an increasingly online gambling ecosystem.

### Hypothesis development

As online gaming environments increasingly incorporate gambling-like features, the boundary between gaming and gambling has become increasingly blurred. As previous established, certain online gaming mechanics mimic the structural and psychological design of gambling [14,16,17]. These embedded mechanics are not merely cosmetic; they are designed to engage players in decision-making under uncertainty, often involving real or virtual currency. Research has suggested that repeated exposure to these features may normalize risk-taking behavior and increase the likelihood of gambling participation [15,19].

Several empirical studies support the notion that online gaming can function as a gateway to gambling. Macey and Hamari found strong associations between esports spectatorship, betting on esports matches, and broader gambling behaviors, highlighting the potential for gaming ecosystems to facilitate gambling entry points [16]. Similarly, Zendle et al. demonstrated a positive relationship between loot box expenditures and problem gambling severity, suggesting that monetary involvement in randomized gaming outcomes may condition users to view gambling as a natural extension of gaming [36]. These findings point to a growing convergence between online gaming and gambling environments, driven by overlapping behavioral incentives and reward structures.

However, much of the existing literature has paid limited attention to how online gaming experience shapes behavior among existing offline gamblers. Individuals with prior offline gambling experience may find online gambling particularly attractive due to its structural similarities with gaming environments. Both domains feature real-time interaction, anonymity, digital payment systems, and strategic elements involving probabilities and decision-making. Familiarity with these features through online gaming may reduce psychological barriers to online gambling adoption and enhance user comfort with digital interfaces. Given these commonalities, it is reasonable to hypothesize that individuals with online gaming experience are more likely to migrate from offline to online gambling platforms. Accordingly, this study posits the following hypothesis:

**H1**: Individuals with prior online gaming experience are more likely to engage in online gambling.

As discussed, financial involvement within online games—through purchases such as loot boxes and randomized rewards—can be linked to intensified gambling behavior. Spending money in online games often involves probabilistic returns or uncertain value, resembling betting behavior in both form and psychological effect. Zendle et al. provide compelling evidence that loot box spending is directly associated with increased problem gambling severity [17]. Other studies suggest that in-game purchases can erode psychological resistance to financial risk, fostering spending behaviors that may carry over into gambling contexts [19,37]. The transition from low-stakes virtual spending to real-money betting may be particularly seamless for individuals accustomed to making regular financial commitments in gaming environments. Based on this reasoning, we propose a second hypothesis:

**H2**: Higher spending on online gaming is positively associated with a greater willingness to engage in online gambling.

## Method

### Sample

To investigate the relationship between online gaming experience and the transition of offline gamblers to online gambling platforms, we utilize survey data collected in South Korea in 2021. The timing of data collection—prior to the full legalization of online horse racing betting in South Korea—offers a valuable opportunity to explore potential consumer demand before policy implementation. The survey was designed to measure public attitudes toward online gambling, particularly in the context of legalized horse race betting. It was administered by a professional online research firm, Embrain, which is one of the leading research service provider listed in the Korea Exchange, the country’s primary securities exchange. Established in 2005, the Korea Exchange is a public institution that oversees South Korea’s major capital markets, including the KOSPI and KOSDAQ, and operates trading platforms for bonds, derivatives, and ETFs within the financial system.

The respondent pool was designed to ensure representativeness across gender, age, and region, and included individuals who consented to the survey’s topic (i.e., online gambling legalization) and research purpose. Respondents were asked about their willingness to engage in online gambling, anticipated gambling frequency, and expected monetary expenditure if online betting became legal. The survey also captured motivations for supporting online gambling legalization, prior gaming experience, and a range of sociodemographic indicators. A total of 742 adults participated in the survey, of whom a subset reported prior offline gambling experience, which forms the analytic sample for this study.

### Ethics statement

The survey is conducted online and only the respondents who consent to the following statement responded to the questions: “This survey is conducted to gather data on public perceptions regarding the introduction of online sales for legally authorized gambling businesses in Korea and to develop appropriate policy measures. The responses collected will be used solely for statistical analysis and will never be used for any other purpose. By participating in this survey, you are providing your consent to take part in the study with full understanding of its purpose.”

### Measures

S1 Appendix defines the variables used in the analysis. The study focuses on three outcome variables that reflect different dimensions of potential engagement with online gambling. The first is Willingness to Gamble Online, a binary indicator equal to one if the respondent would participate in online gambling were it legally permitted, and zero otherwise. Respondents were asked: “If legal horse racing gambling were available online, how likely would you be to use online betting services?” The second variable, Online Gambling Frequency, reflects the expected frequency of gambling, operationalized as the number of online gambling occasions per week. This was assessed using the question: “If legal online betting for horse racing gambling were introduced, how frequently would you plan to engage in online betting on these activities?” Respondents selected from four options, which are assigned the following numerical values: “at least once per day” as 1, “at least once per week” as 1/7, “at least once per month” as 1/30, and “none” as 0. The third outcome, ln(Monthly Online Gambling Expenditure), is the natural logarithm of respondents’ anticipated monthly spending on online gambling. Respondents were given eight spending categories to choose from: (1) less than 10,000 KRW; (2) 10,000–99,999 KRW; (3) 100,000–499,999 KRW; (4) 500,000–999,999 KRW; (5) 1–1.99 million KRW; (6) 2–4.99 million KRW; (7) 5–9.99 million KRW; and (8) 10 million KRW or more. To construct the variable, we assigned the midpoint of each range as the representative value, while for the top category (over 10 million KRW), we assigned a value of 15 million KRW. The logarithmic transformation is applied to account for the skewness typically observed in expenditure data. It is based on the question: “If legal online betting for horse racing gambling were introduced, how much would you plan to spend on online racing bets per month on average?”

The key independent variables relate to respondents’ exposure to online gaming environments. The first is *Online Gaming Experience*, a binary indicator that captures whether a respondent has previously engaged in online gaming. This is based on the question: “Do you currently participate in online games on PC or mobile platforms?” While this measure reflects general gaming familiarity, it may not fully capture exposure to gambling-like elements embedded in game design. To address this, we include *ln(Monthly Online Gaming Expenditure)*, which reflects the monthly financial investment made in online gaming and serves as a proxy for the intensity of engagement with monetized gaming features, including those that resemble gambling mechanics, such as loot boxes or random rewards. Respondents were asked: “On average, how much do you spend per month on the online games you participate in?” They were given eight spending categories to choose from: (1) less than 10,000 KRW; (2) 10,000–99,999 KRW; (3) 100,000–499,999 KRW; (4) 500,000–999,999 KRW; (5) 1–1.99 million KRW; (6) 2–4.99 million KRW; (7) 5–9.99 million KRW; and (8) 10 million KRW or more. To construct the variable, we assigned the midpoint of each range as the representative value, while for the top category (over 10 million KRW), we assigned a value of 15 million KRW. In our sample, none of the respondents selected the top two categories—(7) and (8).

To isolate the effects of gaming experience on gambling intentions, the models control for a set of demographic and psychosocial characteristics that may confound the observed relationships. These include *Age*, *Gender*, *Marital Status*, *Education Level*, *Monthly Income*, *Perceived Health Status*, and *Happiness*. The question on *Perceived Health Status* used a five-point Likert scale with the following options: (1) not healthy at all; (2) relatively unhealthy; (3) neutral; (4) relatively healthy; and (5) very healthy. This variable is transformed using one-hot encoding, with “Not healthy at all” serving as the reference category. Similarly, *Happiness* is assessed on a five-point scale and encoded in the same manner, using “Not happy at all” as the base case. Including these variables helps mitigate bias stemming from individual differences in socioeconomic conditions, emotional well-being, and lifestyle factors that are known to influence gambling behavior.

In an additional analysis, we examine the motivations behind support for the legalization of online gambling, particularly among high-spending online gamers. Understanding these motivations can offer valuable insights into the behavioral drivers that may reinforce or justify gambling participation. To capture this, respondents were asked: “If you agree with the expansion of legal gambling industries into the online space, what is the main reason for your support?” They were given five response options, reflecting distinct motivations: the ability to gamble anytime and anywhere (*Anywhere Anytime*), the appeal of strategy-driven betting (*Data-Based Gambling*), privacy (*Anonymity*), seamless financial transactions (*Convenient Money Management*), and the use of digital tracking systems (*Data-Based User Management*). Respondents were allowed to select one or up to two options.

### Statistical analysis

As part of our analytic procedure, we begin by calculating descriptive statistics, including mean, standard deviation, minimum, and maximum values. These descriptive patterns provide basic insights into respondents’ engagement with online gaming and online gambling. For the main analysis, we employ an ordinary least squares (OLS) linear regression model. Our objective is to examine the associations between key variables, rather than to develop a model optimized for probability prediction. The primary model specification is as follows:


$$\begin{array}{c} Willingness\;to\;Gamble\;Online_{i}=\;\beta_{0}+\beta_{1}Online\;Gaming\;Experience_{i}\;or\\ {\text{ln}\;(Monthly\;Online\;Gaming\;Expenditure)}_{i}+X_{i}\beta +\varepsilon_{i} \end{array}$$ (1)

The coefficient of interest, $\beta_{1}$, captures the effect of prior online gaming experience on the willingness to gamble online when it is legalized. In addition, using a subsample of respondents with prior online gaming experience, we examine the intensity of engagement in online game—proxied by monthly expenditure on online games—to assess whether stronger online gaming behavior predicts a higher willingness to engage in online gambling.

To further explore the association between online gaming experience and potentially problematic online gambling behavior, we analyze two additional outcomes: expected online gambling frequency and expected online gambling expenditure. The corresponding model specification is:


$$\begin{array}{c} Online\;Gambling\;Frequency_{i}\;or\text{ ln}(Monthly\;Online\;Gambling\;Expenditure)\\ =\;\beta_{0}+\beta_{1}\;{\text{ln}\;(Monthly\;Online\;Gaming\;Expenditure)}_{i}+X_{i}\beta +\varepsilon_{i} \end{array}$$ (2)

Again, $\beta_{1}$ represents the relationship between the intensity of past online gaming and more frequent or higher levels of online gambling, potentially indicating a behavioral progression. All models (Equations (1) and (2)) control for a comprehensive set of demographic characteristics, including gender, age, marital status, education, monthly income, perceived health status, and happiness.

Finally, we investigate motivations for supporting the legalization of online gambling, focusing on the subsample of respondents who indicate a willingness to participate in online gambling. The model specification is as follows:


$$Dummy\;for\;Motivation\;Category_{i}=\;\beta_{0}+\beta_{1}\;{\text{ln}\;(Monthly\;Online\;Gaming\;Expenditure)}_{i}+X_{i}\beta +\varepsilon_{i}$$ (3)

Equation (3) is estimated five times, each corresponding to a different motivation category: *Anywhere Anytime, Data-Based Gambling, Anonymity, Convenient Money Management, and Data-Based User Management*. The key coefficient, $\beta_{1}$, captures the extent to which the intensity of online gaming is associated with specific motivations for supporting the shift from offline to online gambling. This model also includes the same set of control variables used in the previous regressions.

## Results

### Descriptive statistics

Table 1 presents summary statistics for the sample of offline gamblers, highlighting patterns in their reported attitudes toward online gambling, gaming behavior, and personal characteristics. On average, 32.34% of offline gamblers expressed a willingness to engage in online gambling if it were legalized, indicating a substantial base of latent demand. The mean value for *Online Gambling Frequency* is 0.1362, corresponding to roughly one gambling episode per week among prospective online gamblers. The average value of *ln(Monthly Online Gambling Expenditure)* is 10.7449, which translates to an estimated monthly expenditure of 46,392 KRW. While this suggests relatively modest average spending intentions, the distribution is right-skewed: 18.58% of respondents reported a willingness to spend more than 300,000 KRW per month, indicating the presence of a high-expenditure subgroup. Turning to online gaming behavior, 55.12% of respondents reported prior experience with online gaming, as indicated by a mean *Online Gaming Experience* value of 0.5512. The average *ln(Monthly Online Gaming Expenditure)* is 9.5417, corresponding to an estimated monthly spending of 13,928 KRW. Again, the distribution reveals a long upper tail, with 9.53% of gamers reporting expenditures exceeding 300,000 KRW per month, suggesting that a subset of highly engaged gamers may be at elevated risk of gambling migration.

**Table 1:** Summary statistics.

	# of Obs.	# of Choices per Category (Proportion)	Mean (S.D.)
**Dependent variables:**			
Willingness to Gamble Online	742		0.3234 (0.4681)
Online Gambling Frequency	210		0.1362 (0.2008)
ln(Monthly Online Gambling Expenditure)	240		10.7449 (1.4175)
**Key independent variables:**			
Online Gaming Experience	742		0.5512 (0.4977)
ln(Monthly Online Gaming Expenditure)	409		9.5417 (1.4984)
**Control variables:**			
Gender	742		
Male		410 (44.74%)	
Female		332 (55.26%)	
Age	742		44.6334 (13.2133)
Married	742		
Married		482 (64.96%)	
Not married		260 (35.04%)	
Education	742		
Middle school or less		9 (1.21%)	
High school		136 (18.33%)	
Vocational college		116 (15.63%)	
University		400 (53.91%)	
Graduate school		81 (10.92%)	
Monthly Income	742		
Less than 1m KRW		22 (2.96%)	
Between 1m and 2m KRW		68 (9.16%)	
Between 2m and 3m KRW		126 (16.98%)	
Between 3m and 4m KRW		154 (20.75%)	
Between 4m and 6m KRW		192 (25.88%)	
Between 6m and 8m KRW		102 (13.75%)	
Greater than 8m KRW		78 (10.51%)	
Perceived Health Status	742		
Not healthy at all		11 (1.48%)	
Relatively unhealthy		75 (10.11%)	
Neutral		284 (38.27%)	
Relatively healthy		309 (41.64%)	
Very healthy		63 (8.49%)	
Happiness	742		
Not happy at all		18 (2.43%)	
Relatively not happy		83 (11.19%)	
Neutral		293 (39.49%)	
Relatively happy		309 (41.64%)	
Very happy		39 (5.26%)	

Note: # of Obs. indicates the number of participants who responded to the specific survey question related to each variable.

The demographic profile of the sample is balanced across key indicators. In terms of *Gender*, 55.26% of respondents are male, indicating an approximately even distribution of male and female participants. The average age is around 45 years, with respondents ranging from 20 to 69 years old. Educational attainment is relatively high, with 64.83% of participants holding a bachelor’s degree or higher. Income distribution follows a bell-shaped pattern, with an average monthly income of 4,610,512 KRW. For self-reported health status, most respondents rated their health as neutral or relatively healthy. Similarly, for happiness, the majority reported feeling neutral or relatively happy.

### Online gaming behavior and willingness to gamble online

We begin our analysis by examining the extent to which online gaming behavior predicts respondents’ willingness to engage in online gambling. Table 2 presents the results from a series of regression models assessing the role of both gaming experience and gaming expenditure. In Model 1, *Online Gaming Experience* is positively and significantly associated with *Willingness to Gamble Online* (β = 0.2109, p < 0.01), indicating that individuals with prior exposure to online gaming are more likely to express an intention to participate in online gambling if permitted. This effect remains statistically significant in Model 2 (β = 0.1866, p < 0.01), even after accounting for demographic and psychosocial control variables. Among the covariates included in Model 2, only *Gender* retains statistical significance, suggesting that men are more inclined than women to express a willingness to gamble online, while other factors such as age, marital status, education, income, health, and happiness do not significantly influence gambling intentions.

**Table 2:** Online gaming experience and willingness to gamble online.

	DEP: Willingness to Gamble Online							
Models	(1)		(2)		(3)		(4)	
	Coef.	T-stat.	Coef.	T-stat.	Coef.	T-stat.	Coef.	T-stat.
Online Gaming Experience	0.2109^***^	6.26	0.1866^***^	5.21				
ln(Monthly Online Gaming Expenditure)					0.1060^***^	6.85	0.1047^***^	6.42
Gender			0.1332^***^	3.82			0.0618	1.25
Age			−0.0017	−0.92			0.0002	0.06
Married			0.0047	0.09			0.0096	0.14
Education (ref. Middle school or less)								
High school			−0.1678	−1.04			0.1102	0.52
Vocational college			−0.1043	−0.65			0.1651	0.78
University			−0.1636	−1.03			0.1045	0.5
Graduate school			−0.2487	−1.50			−0.0409	−0.19
Monthly Income (ref. less than 1m KRW)								
btw 1m and 2m KRW			0.0045	0.04			−0.1017	−0.64
btw 2m and 3m KRW			0.0936	0.88			−0.0417	−0.29
btw 3m and 4m KRW			0.0232	0.22			−0.1423	−0.98
btw 4m and 6m KRW			0.0496	0.47			−0.0388	−0.27
btw 6m and 8m KRW			0.0778	0.70			−0.0238	−0.16
Greater than 8m KRW			0.1068	0.95			0.0587	0.39
Perceived Health Status (ref. Not healthy at all)								
Relatively unhealthy			0.1485	0.98			0.1621	0.95
Neutral			0.1586	1.07			0.1611	0.97
Relatively healthy			0.1190	0.80			0.0702	0.42
Very healthy			0.1841	1.16			0.2712	1.5
Happiness (ref. Not happy at all)								
Relatively not happy			−0.0459	−0.37			−0.0185	−0.12
Neutral			−0.0450	−0.37			−0.0135	−0.09
Relatively happy			−0.0250	−0.20			0.0204	0.14
Very happy			−0.0520	−0.36			−0.0260	−0.15
Constant	0.2072^***^	8.28	0.2214	0.93	−0.5931^***^	−3.97	−0.8065^**^	−2.46
R-squared	0.0503		0.1172		0.1034		0.1541	
Model-p	0.0000^***^		0.0000^***^		0.0000^***^		0.0000^***^	
# of obs.	742		742		409		409	

Note: T-statistics are reported in the parentheses. ^*^, ^**^, ^***^ indicate 10%, 5%, 1% of significance levels. # of Obs. indicates the number of participants who responded to all survey questions corresponding to the variables included in each regression model.

To further assess the impact of financial engagement in online gaming, Models 3 and 4 focus on the subsample of respondents with prior online gaming experience. Here, we evaluate whether spending on online gaming predicts gambling intent. The results reveal a consistent and robust relationship. *ln(Monthly Online Gaming Expenditure)* is positively and significantly associated with *Willingness to Gamble Online* (β = 0.1060, p < 0.01 in Model 3; β = 0.1047, p < 0.01 in Model 4). These findings suggest that not only does gaming experience matter, but the intensity of financial engagement within gaming environments further amplifies the likelihood of online gambling participation. Once again, none of the demographic or well-being variables in Model 4 exhibit significant relationships, indicating that behavioral engagement in gaming is a more powerful predictor of gambling intent than background characteristics.

### Online gaming behavior and excessive online gambling

While the decision to engage in online gambling is itself important, understanding whether such behavior translates into excessive or potentially harmful engagement is a critical policy concern. To address this, we next examine whether online gaming behaviors predict greater intensity of online gambling, as measured by gambling frequency and anticipated expenditure. Table 3 reports regression results for *Online Gambling Frequency*. Models 1 and 2 indicate that *Online Gaming Experience* is not significantly associated with gambling frequency (β = −0.0278, p = 0.91 in Model 1; β = −0.0172, p = 0.51 in Model 2). These results suggest that general gaming experience, while predictive of gambling willingness, does not translate into more frequent online gambling among prospective users.

**Table 3:** Online gaming expenditure and online gambling frequency.

Models	DEP: Online Gambling Frequency							
	(1)		(2)		(3)		(4)	
	Coef.	T-stat.	Coef.	T-stat.	Coef.	T-stat.	Coef.	T-stat.
Online Gaming Experience	−0.0278	−0.91	−0.0172	−0.51				
ln(Monthly Online Gaming Expenditure)					0.0326^***^	3.72	0.0299^***^	3.01
Gender			0.0166	0.50			0.0364	0.96
Age			0.0001	0.07			0.0007	0.39
Married			0.0049	0.11			−0.0143	−0.3
Education (ref. middle school)								
High school			0.0684	0.57			0.1080	0.63
Vocational college			0.0687	0.56			0.0701	0.40
University			0.0265	0.22			0.0575	0.34
Graduate school			0.0633	0.5			0.0764	0.42
Monthly Income (ref. less than 1m KRW)								
btw 1m and 2m KRW			0.0643	0.61			0.0814	0.67
btw 2m and 3m KRW			0.0540	0.58			0.0086	0.08
btw 3m and 4m KRW			0.1307	1.43			0.0682	0.65
btw 4m and 6m KRW			0.0368	0.39			0.0225	0.21
btw 6m and 8m KRW			0.0491	0.50			0.0484	0.44
Greater than 8m KRW			0.0120	0.12			−0.0051	−0.05
Perceived Health Status (ref. Not healthy at all)								
Relatively unhealthy			0.0795	0.60			0.0943	0.74
Neutral			0.0944	0.73			0.1300	1.07
Relatively healthy			0.0370	0.28			0.0865	0.69
Very healthy			0.0166	0.12			0.0704	0.53
Happiness (ref. Not happy at all)								
Relatively not happy			0.0179	0.17			0.0167	0.16
Neutral			−0.0012	−0.01			−0.0289	−0.29
Relatively happy			0.0398	0.40			0.0021	0.02
Very happy			0.1393	1.10			0.0803	0.63
Constant	0.1560^***^	6.06	−0.0603	−0.30	−0.2043^**^	−2.26	−0.4207^*^	−1.77
R-squared	0.0040		0.0843		0.0861		0.1515	
Model-p	0.3629		0.7448		0.0003^***^		0.4425	
# of obs.	210		210		149		149	

Note: T-statistics are reported in the parentheses. ^*^, ^**^, ^***^ indicate 10%, 5%, 1% of significance levels. # of Obs. indicates the number of participants who responded to all survey questions corresponding to the variables included in each regression model.

In contrast, *ln(Monthly Online Gaming Expenditure)* shows a significant and positive association with *Online Gambling Frequency* in Models 3 and 4 (β = 0.0326, p < 0.01 in Model 3; β = 0.0299, p < 0.01 in Model 4). This pattern implies that greater financial engagement in gaming corresponds to increased expected gambling frequency. The effect persists even after controlling for demographics, none of which demonstrate statistical significance. These findings indicate that individuals who are more heavily invested in online gaming financially are also more inclined toward frequent gambling activity, potentially reflecting an elevated risk of problematic behavior.

We further explore this relationship in Table 4 by analyzing determinants of expected online gambling expenditure. Similar to the frequency results, *Online Gaming Experience* alone does not significantly predict *ln(Monthly Online Gambling Expenditure* (β = 0.1899, p = 0.94 in Model 1; β = 0.2276, p = 1.05 in Model 2). These results reinforce the conclusion that gaming experience without financial engagement does not strongly predict gambling intensity. However, *ln(Monthly Online Gaming Expenditure)* is once again a significant predictor of gambling expenditure. In Models 3 and 4, it is positively associated with *ln(Monthly Online Gambling Expenditure* (β = 0.4543, p < 0.01 in Model 3; β = 0.4094, p < 0.01 in Model 4), suggesting that greater spending on online gaming is a strong indicator of increased anticipated gambling losses.

**Table 4:** Online gaming expenditure and online gambling expenditure.

Models	DEP: ln(Monthly Online Gambling Expenditure)							
	(1)		(2)		(3)		(4)	
	Coef.	T-stat.	Coef.	T-stat.	Coef.	T-stat.	Coef.	T-stat.
Online Gaming Experience	0.1899	0.94	0.2276	1.05				
ln(Monthly Online Gaming Expenditure)					0.4543^***^	7.86	0.4094^***^	6.35
Gender			0.6721^***^	3.29			0.3880^*^	1.66
Age			−0.0045	−0.43			−0.0003	−0.03
Married			−0.2195	−0.78			−0.2469	−0.83
Education (ref. middle school)								
High school			−1.8865^**^	−2.34			−1.3550	−1.14
Vocational college			−2.2742^***^	−2.8			−1.6560	−1.39
University			−2.0212^**^	−2.54			−1.5602	−1.32
Graduate school			−1.1550	−1.35			−0.9773	−0.8
Monthly Income (ref. less than 1m KRW)								
btw 1m and 2m KRW			0.6333	0.92			0.3472	0.43
btw 2m and 3m KRW			0.5395	0.87			0.3538	0.5
btw 3m and 4m KRW			0.8050	1.31			0.6120	0.85
btw 4m and 6m KRW			0.7408	1.18			0.6747	0.94
btw 6m and 8m KRW			0.8828	1.34			0.6302	0.84
Greater than 8m KRW			0.4525	0.69			0.3566	0.48
Perceived Health Status (ref. Not healthy at all)								
Relatively unhealthy			0.3470	0.39			0.0741	0.09
Neutral			0.1856	0.22			0.1178	0.14
Relatively healthy			0.3777	0.43			0.1135	0.13
Very healthy			0.1089	0.12			−0.0345	−0.04
Happiness (ref. Not happy at all)								
Relatively not happy			−0.1460	−0.23			0.0694	0.11
Neutral			−0.1650	−0.27			−0.2111	−0.33
Relatively happy			−0.4132	−0.67			−0.1066	−0.16
Very happy			−0.1808	−0.24			−0.5016	−0.65
Constant	1.3993^***^	8.20	2.5203^*^	1.91	6.2072^***^	10.48	7.5910^***^	4.75
R-squared	0.0037		0.1368		0.2053		0.2873	
Model-p	0.3486		0.0570^*^		0.0000^***^		0.0000^***^	
# of obs.	240		240		171		171	

Note: T-statistics are reported in the parentheses. ^*^, ^**^, ^***^ indicate 10%, 5%, 1% of significance levels. # of Obs. indicates the number of participants who responded to all survey questions corresponding to the variables included in each regression model.

Demographic factors have limited influence on gambling expenditure. In Model 2, *Gender* is positively associated with gambling expenditure (β = 0.6721, p < 0.01), and this relationship remains marginally significant in Model 4 (β = 0.3880, p < 0.10), indicating that men expect to spend more on gambling than women. Moreover, educational attainment appears negatively associated with gambling expenditure in Model 2, with high school (β = −1.8865, p < 0.05), vocational college (β = −2.2742, p < 0.01), and university degrees (β = −2.0212, p < 0.05) linked to lower expected spending. However, these associations lose significance once online gaming expenditure is included in Model 4, suggesting that gaming behaviors explain a larger share of the variance.

### Motivations for supporting online gambling legalization

Building on the observed association between online gaming expenditure and excessive gambling tendencies, we turn to the question of why high-spending online gamers support the legalization of online gambling. Understanding their motivations provides insight into behavioral drivers that may reinforce or rationalize gambling engagement.

Table 5 presents regression results examining the relationship between *ln(Monthly Online Gaming Expenditure)* and respondents’ motivations for supporting online gambling legalization. The analysis focuses on five self-reported motivations: the ability to gamble anytime and anywhere (*Anywhere Anytime*), the appeal of strategy-driven betting (*Data-Based Gambling*), privacy (*Anonymity*), seamless financial transactions (*Convenient Money Management*), and the use of digital tracking systems (*Data-Based User Management*). The results indicate that high levels of gaming expenditure are positively associated with support for both *Data-Based Gambling* (β = 0.0544, p < 0.05) and *Anonymity* (β = 0.0683, p < 0.01). These findings suggest that high-spending gamers may be motivated by both strategic and privacy-related considerations. Specifically, they may view online gambling as an activity in which skillful or informed decisions—mirroring data-based gameplay—can enhance outcomes. They may also value the discretion that digital platforms offer, aligning with a preference for private or non-stigmatized gambling experiences. Conversely, gaming expenditure is negatively associated with support for *Data-Based User Management* (β = −0.0378, p < 0.05), indicating resistance to regulatory mechanisms that track or constrain user behavior. This opposition may stem from concerns over autonomy, privacy, or fear of imposed limits that disrupt perceived control over gambling behavior.

**Table 5:** Online gaming expenditure and motivations for supporting online gambling legalization.

Models	DEP: Motivations for supporting online gambling legalization									
	Anywhere anytime		Data-based gambling		Anonymity		Convenient money mgmt.		Data-based user mgmt.	
	(1)		(2)		(3)		(4)		(5)	
	Coef.	T-stat.	Coef.	T-stat.	Coef.	T-stat.	Coef.	T-stat.	Coef.	T-stat.
ln (Monthly Online Gaming Expenditure)	−0.0098	−0.41	0.0544^**^	2.44	0.0683^***^	3.18	−0.0093	−0.45	−0.0378^**^	−2.29
Gender	−0.1318^*^	−1.85	−0.0329	−0.49	−0.1184^*^	−1.84	0.0995	1.63	−0.0035	−0.07
Age	0.0066^*^	1.91	0.0069^**^	2.11	−0.0071^**^	−2.24	−0.0035	−1.16	−0.0036	−1.49
Married	−0.1489	−1.49	−0.0021	−0.02	0.1833^**^	2.03	0.0475	0.55	−0.0029	−0.04
Education (ref. middle school)										
High school	0.0822	0.23	−0.7226^**^	−2.16	0.3821	1.19	0.2709	0.89	−0.3869	−1.56
Vocational college	−0.7287^**^	0.43	0.2144	−2.16	0.3522	0.66	−0.3821	1.14	0.0000	−1.53
University	−0.7076^**^	0.43	0.2713	−2.13	0.2798	0.85	−0.4479^*^	0.92	0.0000	−1.82
Graduate school	0.2154	0.59	−0.8541^**^	−2.48	0.2664	0.8	0.3494	1.11	−0.3237	−1.27
Monthly income (ref. less than 1m KRW)										
btw 1m and 2m KRW	0.1156	0.57	0.2522	1.32	−0.0964	−0.53	−0.3623^**^	−2.08	0.0184	0.13
btw 2m and 3m KRW	0.0713	0.41	0.1817	1.12	−0.1566	−1.01	−0.3132^**^	−2.12	0.0119	0.10
btw 3m and 4m KRW	0.1526	0.86	0.2359	1.42	−0.1433	−0.90	−0.3661^**^	−2.41	0.0017	0.01
btw 4m and 6m KRW	0.1576	0.90	0.2152	1.3	−0.0515	−0.32	−0.2950^*^	−1.95	0.0301	0.25
btw 6m and 8m KRW	0.2403	1.25	0.1629	0.9	−0.0928	−0.53	−0.3385^**^	−2.05	0.0149	0.11
Greater than 8m KRW	0.2320	1.26	0.1332	0.77	−0.1147	−0.69	−0.2664^*^	−1.68	−0.0509	−0.40
Perceived Health Status (ref. Not healthy at all)										
Relatively unhealthy	0.1504	0.56	−0.2878	−1.14	0.0327	0.13	−0.0758	−0.33	0.2241	1.20
Neutral	0.1882	0.73	−0.2763	−1.13	0.0913	0.39	−0.1296	−0.58	0.1304	0.72
Relatively healthy	−0.2253	0.66	0.1365	−0.93	−0.1263	0.59	0.0767	−0.57	0.0000	0.43
Very healthy	−0.057	1.29	−0.0420	−0.22	−0.2347	−0.17	−0.0243	−1.00	0.0000	−0.13
Happiness (ref. Not happy at all)										
Relatively not happy	−0.1236	−0.50	0.0771	0.33	−0.0719	−0.32	0.3652^*^	1.71	0.2340	1.35
Neutral	−0.2762	−1.13	0.1808	0.78	−0.2248	−1.01	0.2699	1.28	0.1263	0.74
Relatively happy	0.2074	−0.95	−0.2324	0.88	0.2376	−1.03	0.2188	1.11	0.0000	1.26
Very happy	0.1308	−1.52	−0.1557	0.49	0.2100	−0.61	0.2562	0.87	0.0000	1.30
Constant	0.3361	0.69	0.5869	1.28	0.4304	0.98	0.1547	0.37	0.4266	1.26
R-squared	0.0702		0.1196		0.1258		0.0866		0.1077	
Model-p	0.7903		0.1480		0.1071		0.5490		0.2587	
# of obs.	240		240		240		240		240	

Note: T-statistics are reported in the parentheses. ^*^, ^**^, ^***^ indicate 10%, 5%, 1% of significance levels. # of Obs. indicates the number of participants who responded to all survey questions corresponding to the variables included in each regression model.

## Discussion

Our findings show that individuals who have previously engaged in online gaming are more likely to express a willingness to participate in online gambling, particularly when their gaming involves financial expenditure. Notably, higher spending on online gaming is positively associated with indicators of potentially problematic gambling behavior. These results suggest that familiarity with digital interfaces, coupled with repeated exposure to gambling-like mechanics—such as microtransactions and loot boxes—may increase individuals’ susceptibility to transitioning from gaming to real-money gambling, potentially heightening the risk of excessive or harmful gambling engagement. Notably, most demographic variables—except for gender—are not significant predictors, underscoring the importance of this behavioral factor. These findings suggest that policymakers and public health practitioners should prioritize high-spending gamers when designing harm-reduction interventions, as they are disproportionately likely to engage in risky gambling behaviors.

Importantly, the study also sheds light on the motivational foundations underlying support for online gambling legalization. High-spending online gamers were more inclined to favor legalization based on strategic considerations, such as the appeal of data-driven betting and the desire for anonymity. At the same time, they expressed skepticism toward regulatory mechanisms involving behavioral tracking or data-based user management. This duality highlights a nuanced perspective: while online gamers often welcome technology-mediated gambling interfaces, they are also wary of interventions that may restrict their autonomy or infringe upon perceived privacy. These attitudes reflect broader concerns about digital surveillance and personal data usage in increasingly algorithmic decision environments [38], which are becoming central to both gaming and gambling experiences. Effective public policy targeting this group may require a nuanced approach—one that maintains a sense of user autonomy while embedding safeguards to prevent financial harm, potentially through behavioral nudges [39]. Public education campaigns that challenge the illusion of control [40] and emphasize the role of randomness in gambling outcomes may be especially effective in reshaping expectations among data-oriented gamblers.

Our findings are broadly comparable to the existing literature, particularly the recent review by Ghelfi et al. [10]. According to their synthesis, younger males are more likely to engage in online gambling and to develop gambling-related problems. Our results support the importance of gender, as males are significantly more likely to express willingness to gamble online; however, we do not find a significant effect of age. In terms of psychological factors, previous research suggests that psychological distress is positively associated with problem gambling [41]. In contrast, our analysis shows that self-reported happiness—which can be interpreted as a composite measure of psychological well-being—is not significantly related to online gambling intention. This may indicate that general well-being measures do not capture the specific emotional or cognitive vulnerabilities linked to problematic gambling behavior.

An especially relevant comparison can be made with the study by Pallesen et al. [42], which examined the relationship between video gaming and online gambling. They found that prior video gaming experience was associated with an increased likelihood of trying online gambling, but not with frequent or problematic gambling. Their interpretation is that while gaming familiarity may lower the threshold for entering online gambling, it does not necessarily lead to more intensive engagement. Our study extends this line of inquiry by focusing specifically on online video gaming, which often incorporates gambling-like features such as loot boxes and other randomized reward mechanisms. Consistent with them, we find that online gaming experience is associated with the initiation of online gambling but not with frequency of gambling. Importantly, we go a step further by examining spending behavior within online gaming and show that individuals with higher online gaming expenditure are significantly more likely to engage in frequent and high-stakes online gambling. This finding highlights the value of distinguishing between mere gaming experience and the intensity of monetary involvement, the latter of which appears to be a stronger predictor of gambling severity.

Our empirical study has several limitations. First, we do not explicitly measure problem gambling. Instead, we analyze respondents’ frequency and spending on online gambling as proxies for gambling intensity. Future research could improve upon this by incorporating established diagnostic tools—such as the Problem Gambling Severity Index (PGSI)—to more directly assess gambling problems among individuals actively engaged in online gambling. Second, while our findings highlight spending on online gaming as a key behavioral predictor of intensive online gambling, not all forms of in-game spending may be associated with gambling-related harm. Future studies should consider categorizing different types of spending mechanisms in online gaming—such as loot boxes, season passes, subscriptions, and premium cosmetic items—to examine whether certain monetization strategies are more closely linked to problematic gambling behavior than others.

## Conclusions

This study contributes to the expanding literature on online gambling by identifying prior online gaming experience as a significant and independent predictor of online gambling participation. While existing research has primarily focused on demographic, economic, and psychological determinants of gambling behavior, the present analysis highlights the critical role of behavioral experiences within online environments. To the best of our knowledge, this is the first empirical study to document the role of online gaming experience in facilitating the transition from offline to online gambling.

The policy implications of these findings are substantial. As online gambling markets continue to expand—particularly in jurisdictions like South Korea where recent legal reforms have opened new avenues for online betting—there is an urgent need to anticipate and address emerging behavioral risks associated with digitally mediated gambling. Recognizing that online gaming can serve as a behavioral precursor to gambling participation allows for more targeted and preventive interventions. Regulatory bodies and gambling service providers should consider implementing consumer protection measures that account for prior gaming experience, such as preemptive spending limits, personalized feedback mechanisms, and educational campaigns that challenge the perceived efficacy of data-driven gambling strategies. These efforts may be particularly important for high-spending gamers who display both elevated gambling intentions and a preference for technological autonomy.

Ultimately, this study advances our understanding of how online leisure activities interact with gambling behaviors in a rapidly evolving online ecosystem. By documenting the experiential and motivational pathways linking online gaming and gambling, the findings provide valuable insights for scholars, practitioners, and policymakers seeking to promote responsible gambling practices in the digital age. Further research is warranted to explore these dynamics across different cultural contexts and platform types, as well as to evaluate the effectiveness of interventions tailored to the unique risk profiles of online video gamers transitioning into online gamblers.

## Supporting information

S1 Appendix Variable definitions. (DOCX)
