# Supplementary material for: The impact of prior online gaming experience on the migration of offline gamblers to online gambling platforms
Source: PLoS One. 2025 Sep 5;20(9):e0331451. doi: 10.1371/journal.pone.0331451 (PMC12412940; doi:10.1371/journal.pone.0331451)
Supplement: S1 Appendix — (DOCX) [file pone.0331451.s001.docx]

**Appendix A. Variable definitions**

|  | **Definition** |
| --- | --- |
| **Dependent variables:** |  |
| Willingness to Gamble Online | This dummy variable represents a respondent’s willingness to participate in online betting if legalized, where 1 indicates willingness and 0 indicates unwillingness. |
| Online Gambling Frequency | For respondents who intend to gamble online, this discrete variable measures how frequently they would engage in online betting if legalized. The responses are assigned numerical values: “at least once per day” as 1, “at least once per week” as 1/7, “at least once per month” as 1/30, and “none” as 0. |
| ln(Monthly Online Gambling Expenditure) | For respondents who intend to gamble online, this variable measures their anticipated average monthly expenditure on online betting if legalized. To account for skewness in the distribution of reported expenditures, the values are transformed using the natural logarithm. |
|  |  |
| **Key independent variables:** |  |
| Online Gaming Experience | This dummy variable indicates whether a respondent has online gaming experience. Those with experience are coded as 1, while those without experience are coded as 0. |
| ln(Monthly Online Gaming Expenditure) | For respondents who have online gaming experience, this variable measures their average monthly expenditure on online gaming. To account for skewness in the distribution of reported expenditures, the values are transformed using the natural logarithm. |
|  |  |
| **Control variables:** |  |
| Gender | This variable indicates gender, coded as 1 for male respondents and 0 for female respondents. |
| Age | This variable represents the respondent’s age. |
| Married | This variable indicates marital status, coded as 1 for married individuals and 0 for not married individuals. |
| Education | This categorical variable represents the highest level of formal education completed. The categories include middle school graduate or less, high school graduate, vocational college graduate, university graduate, and graduate school graduate. |
| Monthly Income | This categorical variable represents monthly income. The categories include less than 1m KRW, between 1m and 2m KRW, between 2m and 3m KRW, between 3m and 4m KRW, between 4m and 6m KRW, between 6m and 8m KRW, and greater than 8m KRW. (m: million) |
| Health Status | This categorical variable measures respondents’ self-assessment of their current health. The five categories are “Not healthy at all,” “Somewhat unhealthy,” “Neutral,” “Somewhat healthy,” and “Very healthy.” |
| Happiness | This categorical variable measures respondents’ self-assessment of their overall happiness. The five categories are “Not happy at all,” “Somewhat unhappy,” “Neutral,” “Somewhat happy,” and “Very happy.” |
